# EDTA enhances antimicrobial activity of PR-39 and Protegrin-1 antimicrobial peptides against carbapenem-resistant *Pseudomonas aeruginosa* in serum

**DOI:** 10.1007/s12223-026-01521-2

**Published:** 2026-05-28

**Authors:** Lukáš Vacek, Antonín Pavelka, Břetislav Lipový, Jana Brtníková, Petra Straková, Edita Jeklová, Marko Šefranko, Dominika Polaštík Kleknerová, Frederik Volný, Lubomír Janda, Lucy Vojtová, Filip Růžička

**Affiliations:** 1 Department of Microbiology, Faculty of Medicine, Masaryk University and St. Anne’s University Hospital, Pekařská 53, Brno, 602 00 Czech Republic; 2 Infectious Diseases and Preventive Medicine, Veterinary Research Institute, Hudcova 296/70, Brno, 621 00 Czech Republic; 3 National Centre for Biomolecular Research, Faculty of Science, Masaryk University, Kamenice 5, Brno, 625 00 Czech Republic; 4 Department of Burns Medicine, Third Faculty of Medicine, Charles University, University Hospital Královské Vinohrady, Šrobárova 50, Prague, 100 34 Czech Republic; 5 Advanced Biomaterials Group, Central European Institute of Technology, Brno University of Technology, Purkyňova 656/123, Brno, 612 00 Czech Republic

**Keywords:** PR-39, Protegrin-1, EDTA, Chitosan, CRPA, Carbapenem-resistant *Pseudomonas aeruginosa*

## Abstract

Carbapenem-resistant *Pseudomonas aeruginosa* represents a frequent and clinically challenging pathogen responsible for both acute and chronic infections. Antimicrobial peptides are emerging as a promising class of novel therapeutic agents due to their broad-spectrum activity, rapid bactericidal activity, and low potential to induce antimicrobial resistance. The antimicrobial efficacy of these peptides can be further enhanced by EDTA. However, their activity is often reduced in the presence of serum, which contains multiple inhibitory components. In this study, EDTA fully restored the antimicrobial activity of PR-39 and Protegrin-1 in serum. Moreover, incorporation of chitosan increased the antimicrobial efficacy of the PR-39/Protegrin-1/EDTA formulation. These findings demonstrate that PR-39, Protegrin-1, EDTA, and chitosan can act synergistically, supporting the feasibility of integrating these components into a unified therapeutic platform.

**Supplementary information:**

The online version contains supplementary material available at https://doi.org/10.1007/s12223-026-01521-2.

## Introduction

Multidrug-resistant (MDR) and extensively drug-resistant (XDR) *Pseudomonas aeruginosa* represents a frequent and challenging pathogen causing acute and chronic infections both within hospital environment and outside (del Barrio-Tofiño et al. 2020). In 2024 update, The World Health Organization designated carbapenem-resistant *P. aeruginosa* (CRPA) a “high” priority pathogen showing increasing trends in resistance, uniquely difficult to prevent, highly transmissible and for which there are few potential treatments (World Health Organization 2024).

The global rise of antimicrobial resistance has intensified the search for novel therapeutic agents, with antimicrobial peptides (AMPs) emerging as a promising class due to their broad-spectrum activity, rapid bactericidal activity, and low potential to induce antimicrobial resistance (Ali et al. 2025). However, translating the promising in vitro activity of AMPs into clinically effective antimicrobials agents remains challenging, largely because their activity is often attenuated in complex biological matrices such as serum (Deslouches et al. 2005).

PR-39 and Protegrin-1 (PG-1), compared to other AMPs, possess unique molecular architectures, which confer greater resilience in biological environments. PG-1 creates a rigid structure consisting of two β-sheets stabilized by two disulfide bridges. PR-39 is characterized by a high proline and arginine content and lacks a conventional secondary structure (polyproline helix II). These properties make them less susceptible to recognition and cleavage by common proteases (Nguyen et al. 2011; Veldhuizen et al. 2014).

Further, it has been proven that sequestration of divalent cations, e.g., by EDTA, can help increase the antimicrobial activity of several AMPs (Maisetta et al. 2017, 2019; Umerska et al. 2018), antibiotics (Lambert et al. 2004), or natural compounds, including chitosan (El-Sharif and Hussain 2011; Abdel-Rhman et al. 2020) in *P. aeruginosa*.

This short report aims to evaluate the effectiveness of EDTA addition on the antimicrobial activity of PR-39 and PG-1 against CRPA strains in the presence of serum as a representative of complex biological matrix and assess the compatibility with chitosan which is an example of the common drug-delivery system.

## Materials and methods

### Bacterial strain selection and characteristics, chemicals

A total of 19 *P. aeruginosa* strains with verified carbapenemase production (according to EUCAST guidelines (The European Committee on Antimicrobial Susceptibility Testing 2017) were tested. Strains designated FF2, FF3, FF8, FF14, FF29, FF36, FF39, FF45, and FF46 were provided by Prof. Ing. Jaroslav Hrabák, Ph.D. (Faculty Medicine in Pilsen, Charles University, Czech Republic). The strains FF73, FF74, FF76, FF79, FF80, FF82, FF84, FF86, and FF87 were isolated from clinical material of patients with wound infections. All strains are stored in the Collection of microorganisms at the Department of Microbiology, St. Anne’s University Hospital and Faculty of Medicine, Masaryk University, Brno, Czech Republic.

Synthetic peptides PG-1 and PR-39 were purchased from MedChemExpress, Sweden. Chitosan with low viscosity (M_w_ of approx. 250 kDa, degree of deacetylation ≥ 75%) was purchased from Sigma-Aldrich (Darmstadt, Germany). Fetal bovine serum was purchased from Merck KGaA (Germany), EDTA-disodium from SERVA (Germany).

### Minimal bactericidal concentration determination of PR-39 and PG-1

The antimicrobial activity of AMPs against CRPA was assessed using the AMIT (antimicrobial inhibition test) Spot-Assay. Briefly, overnight cultures grown in TSB were washed three times with PBS (centrifuged at 3000 rpm, 5 min) and resuspended to achieve a turbidity corresponding to approximately 10^8^ CFU/mL. This suspension was further diluted in PBS to a final working concentration of 10^6^ CFU/mL. The assay was performed in polypropylene tubes by mixing 15 µL of the bacterial suspension with 15 µL of peptides diluted serially (two-fold dilutions ranging from 0.39 to 50 µg/mL); sterile PBS served as a negative control. Samples were incubated at 37 °C for 1 h with shaking (150 rpm).

Subsequently, bacterial suspensions were quantified by ten-fold serial dilutions, plating 10 µL aliquots onto Mueller-Hinton Agar (MHA; Thermo Fisher Scientific Inc., US), and counting colonies after 24 h incubation at 37 °C. The minimal bactericidal concentration (MBC) was defined as the lowest peptide concentration that caused ≥ 3-log (99.9%) reduction in CFU/mL.

### Antimicrobial synergy testing of PR-39 and PG-1 in the presence of EDTA

Based on the determination of PR-39 and PG-1 minimal bactericidal concentration, *P. aeruginosa* FF2 was selected as a representative strain. First, the antimicrobial synergy of each AMP alone and EDTA was tested using a checkerboard assay. The assay was performed by mixing equal volumes (15 µL) of the bacterial suspension, selected peptide, and EDTA. Peptides were diluted serially to obtain a final concentration gradient ranging from 0.008 to 16 µg/mL. EDTA was diluted to a concentration range of 0.39 to 25 mmol/L. The bacterial suspension corresponded to a concentration of 5 × 10^5^ CFU/mL. Sterile deionized water (ddH_2_O) served as a negative control for both AMPs and EDTA. Concentrations of AMPs, EDTA and bacterial suspension stated above were present in tubes after mixing all components (i.e., peptides, EDTA, and bacterial suspension).

### Assessment of the antimicrobial synergy of PR-39 and PG-1 in the presence of EDTA and fetal bovine serum

Antimicrobial synergy testing of PR-39 and PG-1 using checkerboard assay was performed in the presence or absence of EDTA and/or fetal bovine serum (FBS). More specifically, this synergy was tested without EDTA and FBS, with EDTA alone, with FBS alone, and in the presence of both EDTA and FBS.

Similarly to the methodology described in the previous section, the assay was performed by mixing equal volumes (15 µL) of the bacterial suspension, PR-39, PG-1, EDTA, and FBS. Peptides were diluted serially to obtain final a concentration gradient ranging from 0.25 to 16 µg/mL. EDTA was used at a concentration of 6 mmol/L, and the FBS concentration reached 20%. The bacterial suspension corresponded to a concentration of 5 × 10^5^ CFU/mL. Sterile deionized water served as a negative control for both AMPs, EDTA, and FBS.

### Assessment of the compatibility of PR-39, PG-1, EDTA, and chitosan in the presence of fetal bovine serum

To evaluate the compatibility of PR-39, PG-1, EDTA, and chitosan, an MBC was assessed in a gradually increasing concentration of FBS in multiple combinations: (1) PR-39, PG-1, and EDTA (2) EDTA alone, (3) chitosan alone, (4) chitosan and EDTA, (5) chitosan, PR-39, and PG-1, and (6) chitosan, PR-39, PG-1, and EDTA. The concentration gradients for these particular combinations are depicted in Fig. 3.

### Statistical analysis

All experiments were performed three times in triplicates. Quantitative data were presented as means ± standard deviation (SD). Mann-Whitney U-tests were performed to analyze the significance of differences between the two groups.

## Results

### Minimal bactericidal concentrations of PR-39 and PG-1 against carbapenem-resistant *P. aeruginosa*

PG-1 MBC against all tested CRPA strains (*N* = 19) ranged from 0.39 to 6.25 µg/mL. The PR-39 exhibits stronger antimicrobial activity related to its concentration, with MBC values ranging from 0.39 to 1.56 µg/mL across the tested CRPA strains. Detailed MBC values are represented in Supplementary Table 1. Since all CRPA strains showed similar antimicrobial susceptibility against tested AMPs, the FF2 strain was used as a representative for further antimicrobial testing.

### Antimicrobial synergy testing of PR-39 and PG-1 in the presence of EDTA

**Fig. 1:**
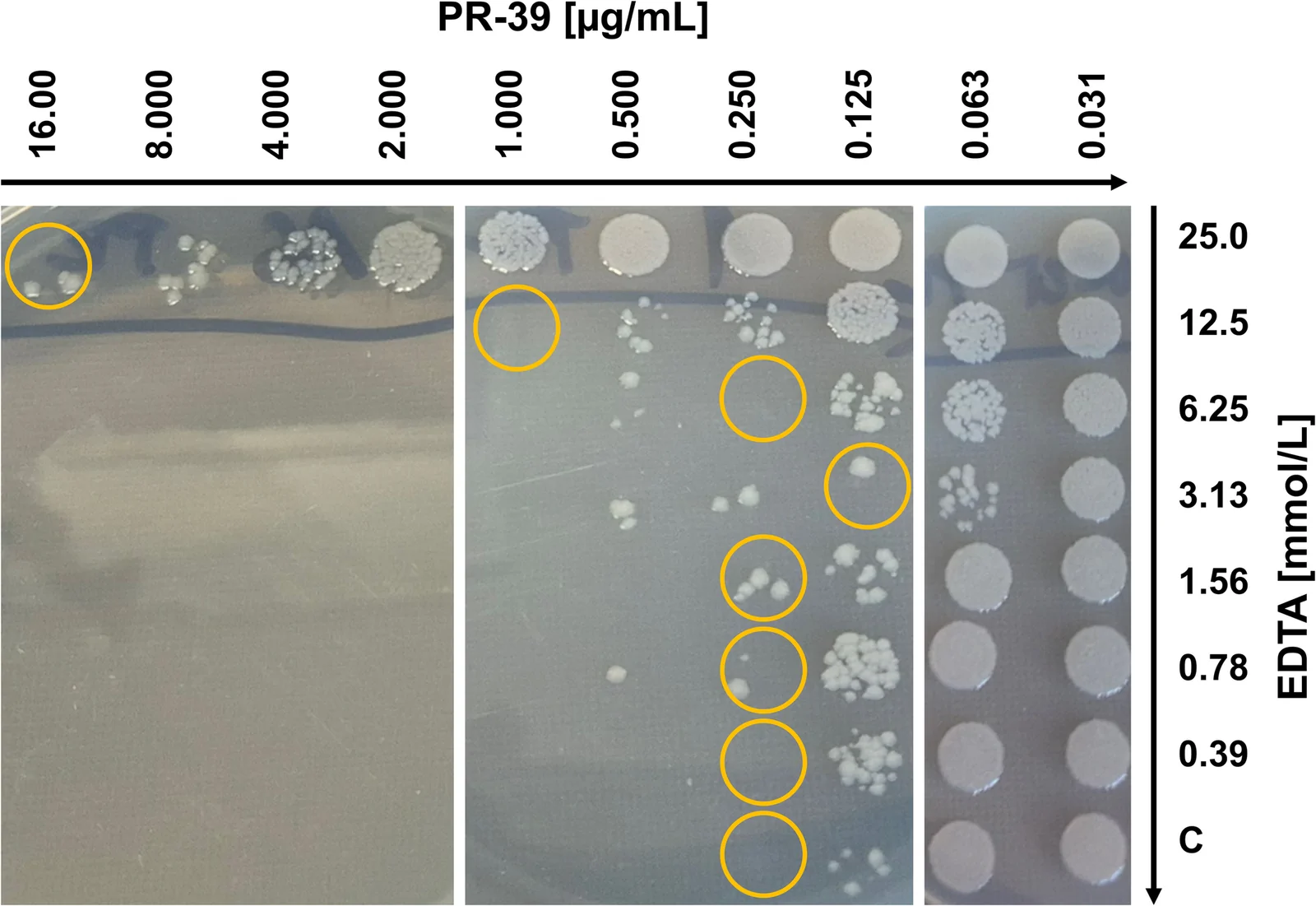
Antimicrobial synergy testing of PR-39 in the presence of EDTA against *Pseudomonas aeruginosa* FF2 after 1 h incubation. The EDTA concentration enhancing the antimicrobial activity of PR-39 is 3.13 mmol/L. Higher concentrations of EDTA displayed decreased susceptibility to both tested peptides. Orange circles denote minimal bactericidal concentration (i.e. <5 bacterial colonies remaining). Please note that the PR-39 concentration gradient was distributed among three separate Petri dishes

**Fig. 2:**
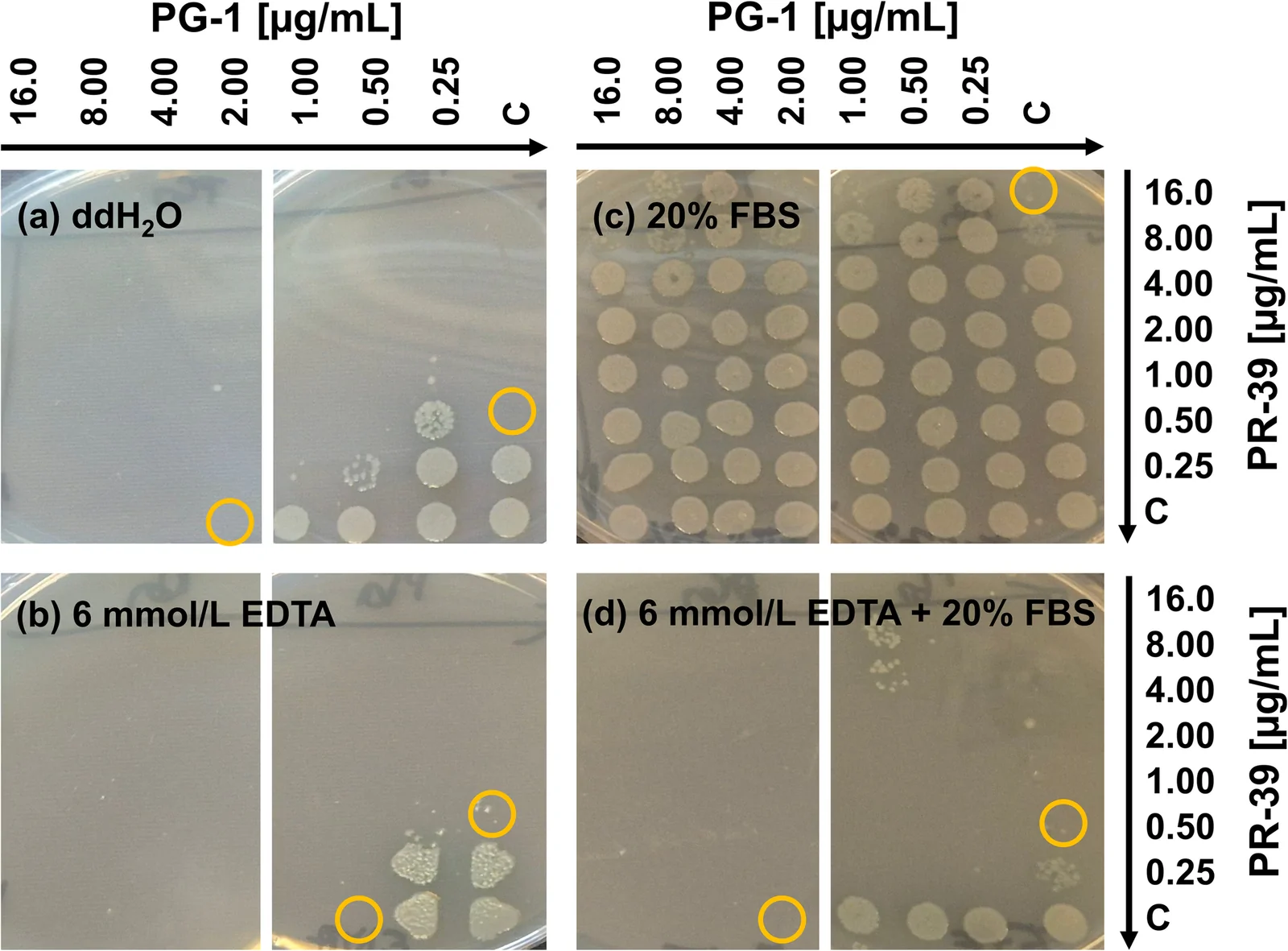
Mutual antimicrobial enhancement of PR-39 and Protegrin-1 (PG-1) in the presence of EDTA and fetal bovine serum against *Pseudomonas aeruginosa* FF2 after 1 h incubation. Checkerboard assay performed in (**a**) ddH_2_O (i.e., without EDTA or FBS), (**b**) with 6 mmol/L EDTA alone, (**c**) with 20% FBS alone, and (**d**) in the presence of both 6 mmol/L EDTA and 20% FBS. Orange circles denote minimal bactericidal concentration (i.e. <5 bacterial colonies remaining). Please note that PG-1 concentration gradient was distributed between two separate Petri dishes

To investigate the bactericidal activity of PR-39 and PG-1 against *P. aeruginosa* FF2 strain, a checkerboard assay was performed. The results show that EDTA concentrations enhancing antimicrobial activity are 3.13 and 6.25 mmol/L for PR-39 and PG-1, respectively. Higher concentrations of EDTA significantly decreased the antimicrobial activity of both PR-39 and PG-1 (*p* < 0.05). Since the results were similar for both peptides, Fig. 1 depicts the results for PR-39 as a representative example. EDTA-enhancing activity for PG-1 can also be observed in Fig. 2 (please compare Fig. 2a and b as discussed in the following section).

### Mutual antimicrobial enhancement of PR-39 and PG-1 in the presence of EDTA and fetal bovine serum

The assessment of antimicrobial potential of the PR-39 and PG-1 combination using checkerboard assay was performed in the four different environments, as described in Fig. 2: (a) in ddH_2_O (i.e., without EDTA or FBS), (b) with 6 mmol/L EDTA alone, (c) with 20% FBS alone, and (d) in the presence of both 6 mmol/L EDTA and 20% FBS. FBS served as a representative of complex biological matrix.

The results clearly show that the presence of serum has a grave detrimental effect on the antimicrobial activity of both tested AMPs. MBC of PG-1 and PR-39 increased 16 times at a minimum (Fig. 2c, *p* < 0.001). On the other hand, the presence of 6 mmol/L EDTA was able to enhance the antimicrobial efficacy of PG-1 four times (Fig. 2b, *p* < 0.05). Most importantly, 6 mmol/L EDTA was able to completely restore the antimicrobial effect of peptides in the presence of 20% FBS (Fig. 2d, *p* > 0.05). In addition, the simultaneous presence of PR-39 and PG-1 displayed an additive antimicrobial effect in ddH_2_O and synergy in the presence of 6 mmol/L EDTA and 20% FBS.

### Assessment of the antimicrobial compatibility of PR-39, PG-1, EDTA, and chitosan in the presence of fetal bovine serum

Since EDTA was previously shown by El-Sharif and Hussain (2011) to increase the antimicrobial activity of chitosan against *P. aeruginosa*, PR-39 and PG-1 were tested for possible compatibility with EDTA and chitosan in the presence of FBS as a representative of complex biological matrix.

Indeed, the evaluation suggested that the addition of EDTA supported the antimicrobial effect of chitosan alone in the presence of FBS. The presence of chitosan, PR-39, and PG-1 without EDTA did not alter the MBC compared with chitosan alone, further confirming the essential role of EDTA.

**Fig. 3:**
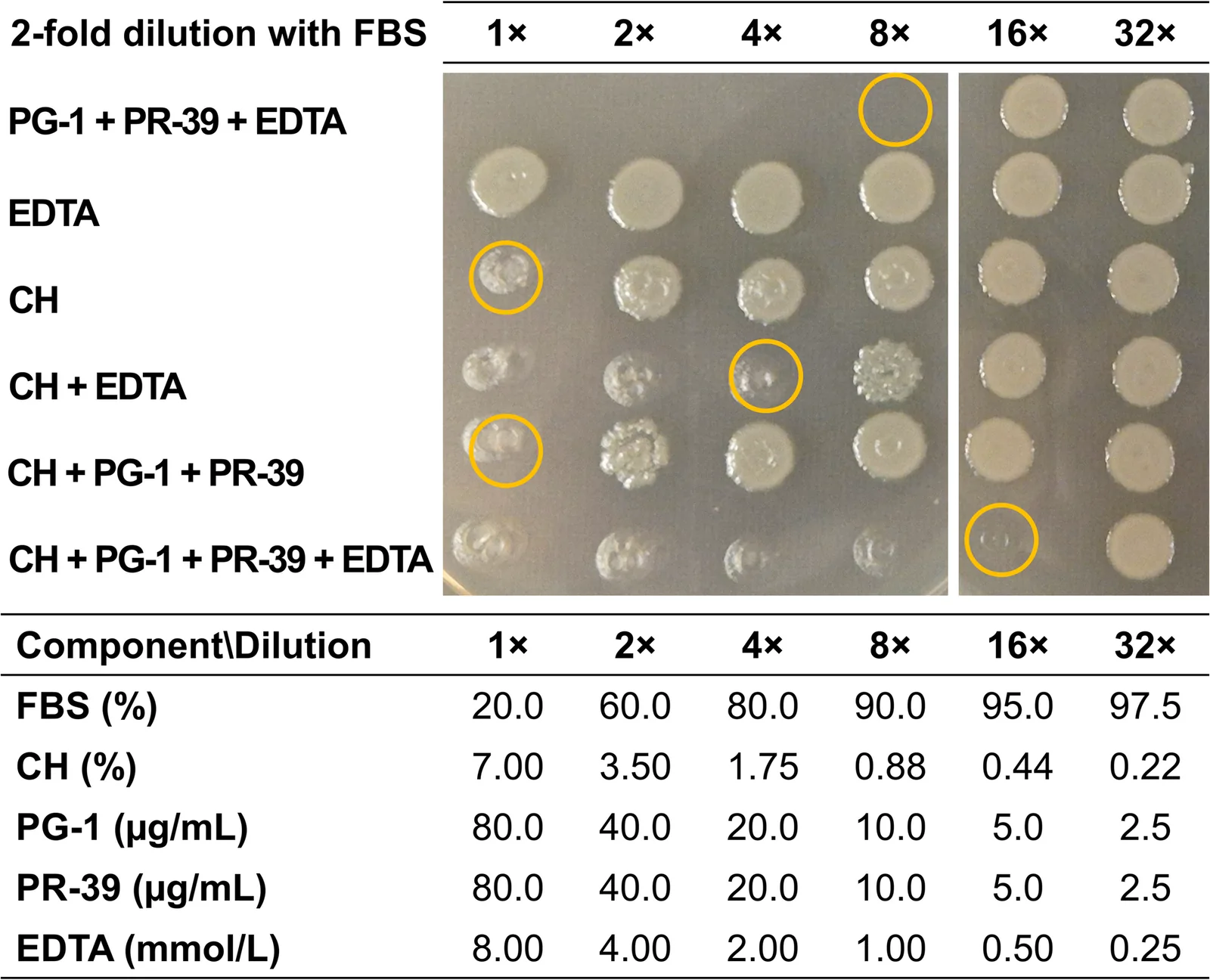
Compatibility of PR-39, Protegrin-1 (PG-1), EDTA, and chitosan (CH) in the presence of fetal bovine serum (FBS). The highest concentrations at the beginning of the dilution series with FBS: PR-39 and PG-1, both at 80 µg/mL, 7% chitosan, 8 mmol/L EDTA. Each combination was diluted with 100% FBS in a 2-fold dilution series. In the upper part, six antimicrobial combinations were tested against *Pseudomonas aeruginosa* FF2 (1-hour incubation). Orange circles denote the MBC (i.e. <5 bacterial colonies remaining) of each combination of components. The lower part lists the corresponding concentrations of each component in the dilution series when present in the particular combination. Please note that dilution series was distributed between two separate Petri dishes

Most importantly, the addition of EDTA to the chitosan/PG-1/PR-39 mixture yielded the most significant antimicrobial effect among all tested combinations (*p* < 0.05). Specifically, the presence of PR-39 and PG-1, both at 5 µg/mL, 0.44% chitosan, and 0.5 mmol/L EDTA, exhibited bactericidal activity against the CRPA strain FF2 in 95% FBS. This implies apparent mutual compatibility among these antimicrobial compounds (Fig. 3).

## Discussion

*P. aeruginosa* is a major opportunistic pathogen causing severe acute and chronic infections. The clinical burden of this organism has intensified over the past two decades, particularly with the rise of multidrug‑resistant strains, including carbapenem‑resistant *P. aeruginosa* (CRPA). In this context, our study sought to determine whether EDTA could enhance the antimicrobial activity of the porcine cathelicidins PR‑39 and PG‑1 against CRPA in the presence of serum, and to assess whether this approach is compatible with chitosan‑based drug delivery systems.

AMPs are central components of innate immunity and exhibit rapid, broad‑spectrum antimicrobial activity. However, despite their promising performance in simplified in vitro systems, their translation into clinically useful therapeutics remains challenging. A major barrier is the significant reduction in AMP activity observed in complex biological matrices (Deslouches et al. 2005).

Previous studies have demonstrated that chelation of divalent cations can enhance AMP activity by disrupting cation‑dependent inhibitory mechanisms (Maisetta et al. 2017, 2019; Umerska et al. 2018). Chelation of Ca²⁺, Mg²⁺ cations by EDTA also destabilizes the outer membrane of Gram-negative bacteria by disrupting lipopolysaccharide cross-bridging. This permeabilizing effect facilitates penetration of AMPs, consistent with the synergistic activity observed in this study (Alakomi et al. 2006). Our findings build on this concept by showing that cation sequestration by EDTA can increase the antimicrobial potential of PR-39 and PG-1 under physiologically relevant conditions (Fig. 2).

In addition, EDTA exhibits pronounced antibiofilm activity against *P. aeruginosa* by chelating Ca²⁺ and Mg²⁺ ions that stabilize the extracellular polymeric matrix. Removal of these ions promotes biofilm dispersal and increases susceptibility to antimicrobial agents (Maroju et al. 2026). Crowther et al. (2025) reported significant biofilm dispersal after treatment with 4% w/v EDTA irrespective of the degree of biofilm maturity, with 56% and 64% biomass reductions in 24- and 48-h *P. aeruginosa* biofilms, respectively.

Importantly, the most abundant protein in the *P. aeruginosa* secretome (at least under laboratory conditions) is an exoprotease termed Pseudomonas elastase B, also designated LasB or pseudolysin (Saint-Criq et al. 2018). LasB is a zinc-dependent metalloenzyme with an additional metal cation (Ca^2+^) as a cofactor in the active site, which has been shown to hydrolyse a variety of host proteins, causing damage to host tissues, disrupting the immune response, and promoting inflammation (Galdino et al. 2019).

Nevertheless, LasB proteolytic and elastolytic activities could be also inhibited by metal chelators. Thus, the addition of EDTA can decreases the virulence potential of *P. aeruginosa* strains and, therefore, indirectly supports wound-healing processes by promoting cell migration and proliferation (Ruffin et al. 2016; Ruffin and Brochiero 2019; Konstantinović et al. 2023).

Beyond the enhancement of AMP activity, EDTA, as a broad-spectrum chelator of divalent cations (Ca²⁺, Mg²⁺, Zn²⁺), functionally inhibits host matrix metalloproteinases (MMPs), since they require divalent cations for catalytic activity (Zn²⁺) and structural stability (Zn²⁺, Ca²⁺) (Laronha and Caldeira 2020). For example, Ollivier et al. (2003). have documented a 99.4% reduction of matrix metalloproteinase MMP-2 and MMP-9 activity in the tear film of horses with active corneal ulcers when treated with EDTA.

Therefore, EDTA inhibits proteolytic degradation of key immune mediators such as complement proteins, cytokines, chemokines, immunoglobulins, and antimicrobial peptides. This decrease in protease activity helps maintain the structural and functional integrity of host immune proteins within protease-rich infection sites, thereby supporting innate immune responses and enhancing the activity of exogenously applied AMPs (Marshall et al. 2017; Yao et al. 2025). Under our experimental conditions, EDTA significantly potentiated the antimicrobial effect of PR-39 and PG-1.

In contrast to the effects discussed above, a reduction in the antimicrobial efficacy of PR-39 and PG-1 was observed at higher EDTA concentrations. Based on available literature, interactions between EDTA and either PR-39 or PG-1 have not been previously reported. Possible explanations for these observations include excessive chelation interfering with peptide–membrane interactions or reducing the bioavailable fraction of peptides, thereby reducing their antimicrobial activity. These observations emphasize the importance of optimizing EDTA concentration to achieve maximal additive value.

Formulations combining AMPs with EDTA may further benefit from appropriate drug-delivery systems, among which chitosan-based matrices are widely used. El-Sharif and Hussain (2011) previously demonstrated that EDTA enhances the antimicrobial activity of chitosan, suggesting potential synergy between these components. This phenomenon was also confirmed in the presented study (Fig. 3). In addition, chitosan exhibits hemostatic activity and promotes adhesion and activation of macrophages, thereby accelerating the onset of the proliferative phase of wound healing. Its intrinsic mucoadhesive character also ensures that formulations containing AMPs and EDTA maintain prolonged contact with the wound bed despite ongoing exudation, particularly when delivered as injectable hydrogels (Feng et al. 2021).

The concentrations of AMPs, EDTA, and chitosan used in this study correspond to ranges commonly applied in antimicrobial formulations for therapeutic applications (Deng et al. 2022; Sahraneshin-Samani et al. 2022; Kim et al. 2023; Gonciarz et al. 2025). Chitosan in chitosan-based hydrogels for skin tissue regeneration and wound healing is usually used in a concentration range of 0.2 to 10%, though minimum inhibitory concentrations of chitosan itself are generally detected in the range of 0.005 to 0.1%, depending on the bacterial strain and molecular weight of chitosan (No et al. 2002; Liu et al. 2006).

EDTA is usually tested in concentrations up to 4% (approx. 100 mmol/L of EDTA disodium salt) (Crowther et al. 2025), though, e.g., commercially available wound care products contain 0.1% EDTA (RescuDerm^®^, NociPharm, Inc., Canada or Biostep^®^ Smith & Nephew Wound Management, Inc., USA) (Sahoo et al. 2026). Moreover, EDTA can be used in chelation therapy, usually administered intravenously as 0.4% solution (2 g of EDTA diluted in 500 mL physiological saline) over 2 h (Fulgenzi and Ferrero 2019).

PR-39 was previously tested at concentrations up to 40 µmol/L (approx. 186 µg/mL of PR-39) for its antimicrobial activity. Veldhuizen et al. (2014) reported minimal bactericidal concentrations of PR-39 in *Escherichia coli* (1.25 µmol/L) and for *Bacillus globigii* at 2.5 µmol/L. In addition, PR-39 was tested in a rat myocardial infarction model together with adrenomedullin, where the combination therapy improved cell viability and cardiac function, attenuated apoptosis and myocardial injury, and promoted angiogenesis (An et al. 2017).

In vivo PG-1 efficacy was tested by Steinstraesser et al. (2001) in a rat model of thermally injured skin infected with *P. aeruginosa*. Using topical application or intradermal injection of a 200 µg/mL PG-1 solution, this study demonstrated that PG-1 is highly effective against clinically relevant isolates from human burn wounds, making it a potential candidate for local treatment of infected burn wounds (Steinstraesser et al. 2001). Xu et al. (2025) showed that PG-1 is also effective against extraintestinal pathogenic *E. coli* in a murine model. Four-week-old female BALB/c mice were treated with 3.2 mg/kg PG-1 (approx. 48 µg per one animal) intraperitoneally administered 1 h post-infection. PG-1 treatment resulted in a higher survival rate compared to the control group.

Previous studies have not reported mutual PR-39, PG-1, and EDTA compatibility with the chitosan-based delivery system. Our results demonstrate (Fig. 3) that these components can act synergistically, supporting the feasibility of combining AMPs, EDTA, and chitosan into a unified therapeutic platform. This approach may enable treatment strategies to be tailored to specific clinical contexts or patient needs and represents a promising direction for future research and potential clinical applications targeting infections caused by antibiotic-resistant bacteria.

## Conclusions

This study demonstrates that EDTA can effectively enhance the antimicrobial activity of the porcine peptides PR‑39 and PG‑1 against CRPA in serum, addressing a key limitation that has hindered the clinical translation of antimicrobial peptides. Our findings reveal that this potentiating effect is concentration‑dependent, highlighting the need for careful optimization of EDTA levels to maximize therapeutic benefit. Furthermore, we showed that PR‑39, PG‑1, and EDTA are mutually compatible with a chitosan‑based delivery system. These results support the integration of antimicrobial peptides, EDTA, and chitosan into a unified therapeutic platform and highlight a promising strategy for treating infections caused by antibiotic-resistant *P. aeruginosa*.

## Supplementary information

Below is the link to the electronic supplementary material.

Supplementary File 1 (DOCX 15.2 KB)

## Data Availability

All data supporting the findings of this study are available within the paper and its Supplementary Information.
