# Supplementary material for: EDTA enhances antimicrobial activity of PR-39 and Protegrin-1 antimicrobial peptides against carbapenem-resistant Pseudomonas aeruginosa in serum
Source: Folia Microbiol (Praha). 2026 May 28;71(5):1485–93. doi: 10.1007/s12223-026-01521-2 (PMC13736336; doi:10.1007/s12223-026-01521-2)
Supplement: Supplementary file 1 [file 12223_2026_1521_MOESM1_ESM.docx]

**Supplementary Table 1.** Minimal bactericidal concentration (MBC) in µg/mL of Protegrin‑1 (PG-1) and PR-39 in phosphate-buffered saline against carbapenem-resistant *Pseudomonas aeruginosa* strains.

| Strain | PG-1 | PR-39 |
| --- | --- | --- |
| FF2 | 3.13 | 0.78 |
| FF3 | 3.13 | 0.39 |
| FF8 | 3.13 | 1.56 |
| FF14 | 3.13 | 1.56 |
| FF29 | 1.56 | 1.56 |
| FF36 | 6.25 | 0.39 |
| FF39 | 0.39 | 0.78 |
| FF45 | 0.78 | 0.78 |
| FF46 | 1.56 | 0.78 |
| FF73 | 0.39 | 1.56 |
| FF74 | 0.78 | 0.39 |
| FF76 | 1.56 | 1.56 |
| FF79 | 3.13 | 1.56 |
| FF80 | 1.25 | 0.78 |
| FF82 | 1.56 | 0.39 |
| FF83 | 6.25 | 0.39 |
| FF84 | 0.78 | 1.56 |
| FF86 | 6.25 | 0.78 |
| FF87 | 6.25 | 0.39 |
